# How innovation can be defined, evaluated and rewarded in health technology assessment

**DOI:** 10.1186/s13561-021-00342-y

**Published:** 2022-01-03

**Authors:** Juan Carlos Rejon-Parrilla, Jaime Espin, David Epstein

**Affiliations:** 1 Área de Evaluación de Tecnologías Sanitarias de la Fundación Pública Andaluza Progreso y Salud (AETSA-FPS), Sevilla, Spain; 2 Andalusian School of Public Health, Granada, Spain; 3 CIBER of Epidemiology and Public Health (CIBERESP), Madrid, Spain; 4 Biosanitary Research Institute (ibs.GRANADA), Granada, Spain; 5 University of Granada, Granada, Spain

**Keywords:** Innovation, Health technology assessment, Value, Health policy

## Abstract

**Background:**

What constitutes innovation in health technologies can be defined and measured in a number of ways and it has been widely researched and published about. However, while many countries mention it as a criterion for pricing or reimbursement of health technologies, countries differ widely in how they define and operationalise it.

**Methods:**

We performed a literature review, using a snowballing search. In this paper, we explore how innovation has been defined in the literature in relation to health technology assessment. We also describe how a selection of countries (England, France, Italy, Spain and Japan) take account of innovation in their health technology assessment frameworks and explore the key methodologies that can capture it as a dimension of value in a new health technology. We propose a way of coming to, and incorporating into health technology assessment systems, a definition of innovation for health technologies that is independent of other dimensions of value that they already account for in their systems, such as clinical benefit. We use Spain as an illustrative example of how innovation might be operationalised as a criterion for decision making in health technology assessment.

**Results:**

The countries analysed here can be divided into 2 groups with respect to how they define innovation. France, Japan and Italy use features such as severity, unmet need and therapeutic added value as indicators of the degree of innovation of a health technology, while England, Spain consider the degree of innovation as a separate and additional criterion from others. In the case of Spain, a notion of innovation might be constructed around concepts of `step-change’, `convenience’, `strength of evidence base’ and `impact on future research & development’.

**Conclusions:**

If innovation is to be used as operational criteria for adoption, pricing and reimbursement of health technologies, the concept must be clearly defined, and it ought to be independent from other value dimensions already captured in their health technology assessment systems.

**Supplementary Information:**

The online version contains supplementary material available at 10.1186/s13561-021-00342-y.

## Background and introduction

There is a huge industry dedicated exclusively to the discovery and development of new and innovative health technologies. The average research and development (R&D) investment per approved new compound is about UD$1,5 billion [1, 2]. In such a competitive industrial environment, it becomes vital to the industry to read any signals public payers may send around what they value and what they do not regard as relevant when it comes to deciding which health technologies to fund and at what price. Health Technology Assessment (HTA) is defined by the World Health Organization (WHO) as the approach used to inform policy and decision-making in health care, especially on how best to allocate limited funds to health interventions and technologies [3]. The criteria used to judge what constitutes desirable health interventions and technologies can vary amongst HTA systems depending on their aims and the methodologies picked to reach them.

This paper considers how innovation is defined, evaluated and rewarded in HTA. The term is widely used and encompasses multiple attributes. Most HTA systems evaluate features of innovation that consider the impact of a product from the perspective of current patients (therapeutic benefit, unmet need, safety, administration) or current budget holders (cost), also called the “static” perspective [4]. Examples of this approach can be seen in the paper published by de Solà-Morales et al. [5], which looks at how innovation is defined from a current payer’s perspective, or also in the work led by Karl Claxton on the cost-effectiveness threshold that defines the opportunity cost of decisions on new technology in terms of the marginal health displaced in the current NHS [6]. HTA systems less frequently explicitly consider the “dynamic” consequences or incentives created by a decision to adopt or not a new technology on the direction of future R&D and ultimately, further innovations. These terms overlap to some extent with the idea of the source of innovation being `pulled by demand’ or `pushed by supply’ or entrepreneurship [7].

Previous reviews in this topic have explored specific aspects of innovation: from an organizational point of view [8], for medicines [5, 9–11] and for medical devices [7]. However, none look at the question in a holistic way to consider how innovation should be included as a criterion for HTA in practice.

Hence, the overall aim of this article is to construct a broad concept of innovation and a process of tailoring it to individual HTA systems that can be useful for healthcare policy makers considering if and how their HTA frameworks capture innovation. To fulfill this aim, we followed three objectives: First, to assess with reference to the literature the theoretical justification for which attributes of innovation ought to be considered in HTA. Second, to assess how HTA bodies in France, Italy, England, Spain and Japan consider these issues in their assessments for adoption or pricing & reimbursement (P&R). Finally, Spain is taken as a case-study to consider how the degree of innovation should and can be strengthened in HTA decisions, and we discuss the relevance of the findings for other HTA systems.

## Methods

We performed a literature review, using a snowballing search [12]. We chose this technique because the literature suggests it is a more effective approach for complex and heterogeneous evidence than more formal protocol-driven searches [13]. The steps in a snowball search are: 1) Establish the research question and inclusion and exclusion criteria 2) Identify the start set: a small number of seminal papers or highly cited papers 3) Backward snowballing: Reviewing the reference lists of the seminal papers 4) Forward snowballing: Searching for papers that cite the seminal papers.

Our inclusion criteria were that the papers included dealt with the concept of innovation in HTA decisions (adoption, reimbursement or pricing) about all types of health technologies (medicines, devices and diagnostics). We excluded: 1) papers where “innovation” was used as a term to refer exclusively to therapeutic benefit or similar terms, already separately accounted for in HTA; 2) papers that did not add anything new on top of the seminal papers; 3) papers that focused on concepts of organizational innovation that are not relevant to HTA adoption or P&R decisions; 4) editorials; 5) regulatory approval criteria and literature that focus exclusively on efficacy, safety and quality. We included papers both in English and Spanish. There was no limitation on the dates when papers were published. One of the authors of this paper made a first selection of included and excluded papers, a second author double checked it and a third author was available to resolve any discrepancies. The search strategy is described in more detail in Additional file 1.

Not all concepts are eligible or useful for decision making. Diaby and Goeree [14] recommended that items need to exhibit all the following properties: ‘value relevance’, ‘understandability’, ‘measurability’, ‘non-redundancy’, ‘independence’ and ‘comprehensiveness’. We use this framework as a test for each feature of innovation identified in the literature, seeking to trim these down to a smaller set of items that jointly display these properties, and could potentially be used as criteria in HTA. We then consider methods that could be used to measure or rank health technologies in practice on the basis of the degree of innovation in the chosen countries. In the end, countries choose the criteria that they feel best align and promote their specific aims. Our intention is to identify those criteria that have some theoretical justification and can be measured.

We also assess how HTA bodies in France, Italy, England, Japan and Spain consider innovation in their assessments for adoption, P&R processes. Our choice of countries is based on our judgment of HTA systems that take different stands on whether and how they account for degree of innovation as an independent source of value of new health technologies. We chose a set of countries that allow us to analyse different approaches to HTA to show how innovation can be embedded in different HTA systems for the evaluation and reimbursement of health technologies. Our reasons for including France and England are that they have internationally leading nationally centralized systems that work following high standards of transparency, one rewarding innovativeness as an independent feature (England) whilst the other entangles the concept more with other criteria (France). Japan presents a recently reformed centrally coordinated HTA system, different to the rest of the countries we will be looking into, in that they reward innovative new technologies by applying a system whereby the technologies considered to be innovative receive a premium price beyond the price of the comparator. Italy, whilst having a national agency, is a more fragmented model, with the added interest of having recently introduced a new method to capture innovation [15]. Spain goes one step further in how decentralized it is in its’ HTA activities, having several regional agencies as well as national entities, each with parallel competencies. The main interest in this country is that the law includes degree of innovation amongst the criteria that should be used to make P&R decisions for drugs [16], but provides no guidance on how to define or measure this concept. Despite the size of R&D investment having been consistently higher in the US compared with Europe, and the US being the biggest pole of clinical trials worldwide [1], we decided not to include the US because P&R decisions in practice are not consistently based around the HTA evidence produced by leading research institutes such as the Institute for Clinical and Economic Review.

## Results

### Literature search

**Fig. 1:**
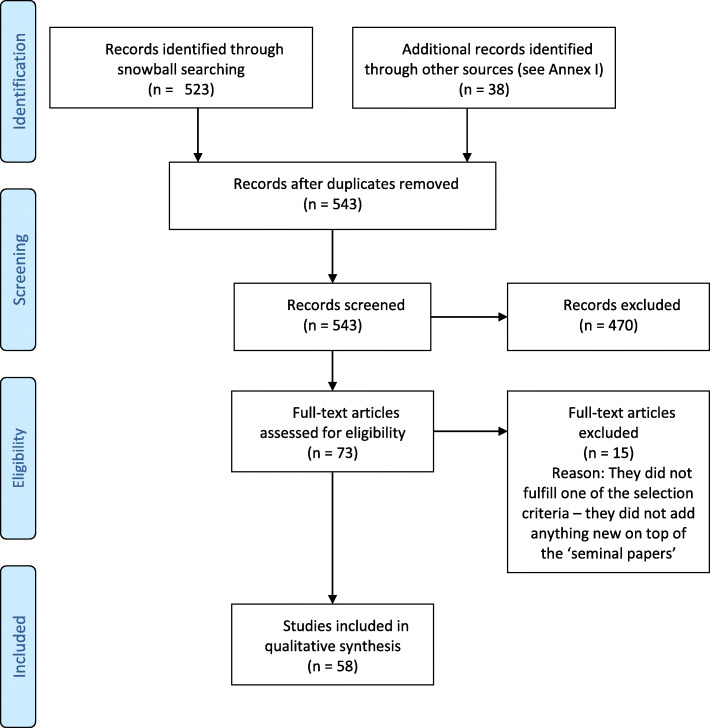
PRISMA flow diagram [58]

The bibliographic search described in Additional file 1 identified 38 papers. From this list, and papers recommended by colleagues and contacts, four seminal papers were chosen [5, 7, 17, 18]. Reference lists of these 4 papers were examined and we used Google Scholar to identify the articles that cited the 4 papers. These forward and backward snowball searches identified 523 papers. Adding in the aforementioned 38 papers and eliminating duplicates provided 543 articles to be screened by title. We reviewed abstracts when titles were not enough to decide. From these, we assessed 73 full papers and decided to exclude 15. That left us with the 58 papers that we included in our review and final synthesis. These are briefly summarised in Additional file 2. Figure 1 below shows the flow diagram.

Table 1 summarises the attributes related to innovation that were discussed in the included papers. All of the concepts of innovation discussed in the 58 papers in the literature search were covered in 5 papers: the four seminal papers [5, 7, 17, 18], and one other [19]. Hence only these papers are included in Table 1.

**Table 1:** Items found in the literature to compose a broad concept of innovation for health technologies

	Solà-Morales et al. (2018)	Angelis & Kanavos (2017)	Ciani et al. (2016)	Garrison et al. (2017)	Mestre-Ferrandiz et al. (2012)
*Attributes related to therapeutic added value of technology, compared to relevant comparator*					
Therapeutic benefit	✓	✗	✓	✗	✓
*Attributes related to step-change*					
Breakthrough status	✗	✗	✓	✗	✗
*Attributes related to the underlying health condition of the patients & current care*					
Availability of existing intervention	✓	✗	✗	✗	✓
Unmet need	✓	✗	✗	✗	✓
*Attributes related to safety*					
Safety	✓	✗	✓	✗	✓
*Attributes related to convenience*					
Patient usefulness (i.e. convenience)	✓	✓	✓	✗	✓
Carer usefulness (i.e. convenience)	✗	✗	✗	✗	✓
Administration	✗	✗	✗	✗	✓
*Attributes related to economic impact*					
Cost or budget impact	✓	✗	✓	✗	✓
Impact on non-healthcare resources and productivity benefits	✗	✗	✗	✗	✓
*Attributes related to evidence base*					
Strength of clinical evidence	✓	✗	✗	✗	✗
Learning curve	✗	✗	✓	✗	✗
*Attributes related to R&D and impact on future innovation pipeline (dynamic effects)*					
Novelty	✓	✓	✗	✗	✗
Spill-over effects	✗	✓	✗	✓	✗
Real option value	✗	✗	✗	✓	✗

For medicines, Solà-Morales et al. (2018) [5] identified 10 dimensions of innovation in the literature which, in order of most to least widely referred to in identified papers, are: therapeutic benefit, novelty (of structure or mechanism of action), availability of existing treatment, unmet need, safety, newness, administration, clinical evidence, cost, and ‘other’.

The Advance Value Framework is a Multiple Criteria Decision Analysis (MCDA) framework for medicines proposed by Angelis & Kanavos (2017) [18]. They do not phrase a definition for innovation as such, but they do include it as one of the 5 dimensions of value that make up their framework. Their proposed notion of innovation captures the following value items: (a) medicine’s mechanism of action, (b) spill-over effects, and (c) patient usefulness (i.e. convenience).

Garrison et al. (2017) [17] include spill-over effects as one of the potential “sources of value” for health technologies. They define it as the knowledge that is produced in the process of coming up and using a particular innovative treatment that spills over to foster other innovations and benefits other patient groups. That is, the adoption of a given product with benefit for a specific group of patients produces what economists refer to as a “knowledge externality”, with spillover benefits for others. Garrison also discussed ‘real option value’. This is the value to a patient of extending their life for a limited period of time because that opens up the possibility for them to benefit from future medical advances, above and beyond the value that the immediate clinical benefit that the intervention brings to the patient.

Ciani and collaborators (2016) [7] identify three broad dimensions of innovation related to medical devices: (i) the source of innovation (demand or supply driven), (ii) the degree of discontinuity introduced (incremental or breakthrough) and (iii) the impact or consequences of innovation (measurable changes in terms of patients’ benefits, quality of the service or costs).

Ciani also discusses the ‘learning curve’ – the issue around how innovations are incorporated into routine practice, and how that can affect the measured performance of the new intervention over time. The learning curve might apply to all health technologies but it is particularly acute for non drug health technologies such as medical devices.

Mestre-Ferrandiz et al. [19] advocate for a concept of innovation that is incremental or a matter of degree, as opposed to it being a quality that is either present or not in a health technology. They characterise innovation for pharmaceuticals using 10 attributes grouped under 3 general headings: (A) Health gains, including: (1) tackling a new disease and/or indication (2); health gains measured in quality of life and/or life duration (3); faster health improvement (4); reduced side-effects and/or improved tolerability (5); reduced negative interactions with other therapies (6); treating better than current standard of care one or more different patient subpopulations; (B)(7) Patients’ / carers’ convenience; (C) Other societal gains, including cost savings: (8) releasing other healthcare resources (9); releasing other non-healthcare resources (10); productivity benefits.

### How innovation is perceived, measured and rewarded in Spain, France, Italy, England and Japan

Payers and HTA bodies across the world use the `degree of innovation` as a criterion for adoption or P&R, though, in parallel with the academic literature, the meaning of this term is not precisely or consistently defined. Table 2 summarises the stated position of HTA bodies in Spain (Interministerial Medicinal Products Pricing Committee – CIPM & the Spanish Agency of Medicines and Medical Devices – AEMPS), England (National Institute for Health and Care Excellence – NICE), Italy (Agenzia Italiana del Farmaco – AIFA), France (Haute Autorité de Santé – HAS) and Japan (National Institute of Public Health – NIPH). Note that some of these institutions also hold other responsibilities than HTA, such as AIFA, which is also responsible for the regulation of medicines in Italy [15]. We classify attributes into 8 dimensions: added therapeutic value, step change, underlying health condition, safety, convenience, economic impact, evidence base, and dynamic impacts that may influence future R&D.

**Table 2:** Criteria for HTA recommendations in England, Italy, France and Spain

	NICE (England and Wales)	CIPM & AEMPS (Spain) (‡)	AIFA (Italy)	HAS (France)	NIPH (Japan)
	All HTA	Medicines	Medicines	Medicines	All HTA
*Attributes related to therapeutic added value of technology, compared to relevant comparator*					
Therapeutic benefit	✓	✓	✓ (I)	✓(I)	✓(I)
*Attributes related to step-change*					
Step-change in the management of the condition	✓(I)	✗	✗	✗	✓(I)
Disruptiveness	✗	✗	✗	✓	✗
Breakthrough status	✗	✗	✗	✗	✗
Demonstratable and distinctive benefit	✓(I)	✗	✓ (I)	✗	✗
*Attributes related to the underlying health condition of the patients & current care*					
Severity of underlying disease	✗	✓	✗	✓(I)	✗
Impact on the health of the population	✓	✗	✓	✓(I)	✓
Availability of existing intervention	✓	✓	✓	✓	✓
Unmet need	✓	✓	✓(I)	✓	✓
*Attributes related to safety*					
Safety	✓	✓	✓	✓	✓(I)
*Attributes related to convenience*					
Administration	✗	✓	✓	✓	✓(I)
Patient usefulness (i.e. convenience)	✓	✗	✓	✓	✓(I)
Carer usefulness (i.e. convenience)	✗	✗	✗	✗	✗
*Attributes related to economic impact*					
Cost or budget impact	✓	✓	✓	✓	✓
Impact on non-healthcare resources and productivity benefits	✗	✗	✗	✗	✗
Incremental cost-effectiveness ratio	✓	✓	✓	C	✓
*Attributes related to the evidence base*					
Strength of clinical evidence	✓	✗	✓(I)	✓	✓
Learning curve	✗	✗	✗	✗	✗
*Attributes related to R&D and impact on future innovation pipeline (dynamic effects)*					
Novelty	✓(I)	✗	✓	✗	✓
Spill-over effects	✗	✗	✗	✗	✗
Real option value	✗	✗	✗	✗	✗
References	[20, 21]	[21–23]	[15, 21]	[21, 24, 25]	[26, 27]

Note: (I) refers to whether the criteria is labeled by the HTA agency as an attribute of ‘innovation’ (C) refers to ‘in certain circumstances’ (‡) Spain has a criteria labeled ‘innovation’ but no definition or further guidance is provided

We used the same broad dimensions in Tables 1 and 2, though some of the items differ. Table 1 is a summary of how the selected literature defines innovation in HTA. For instance, incremental cost-effectiveness ratio is not present in Table 1 because it was not specifically listed in the included papers. Table 2 includes all the items in Table 1, together with the criteria used by the selected HTA bodies to capture innovation in their frameworks. Hence Table 2 shows the degree of alignment of the criteria used by HTA agencies against each other and compared with the academic literature.

In England the Kennedy report (2009) called for NICE to define innovation and for the Department of Health to regularly update their priorities for innovation in the healthcare sector [28]. This would allow stakeholders across the healthcare ecosystem to judge whether new health technologies respond to the declared needs of the system or not. NICE were encouraged to regard innovation as a social value worth pursuing independently for instance from maximizing health outcomes. As a result, NICE established 3 conditions that must be met by health technologies to be classed as innovative [29]:

1. The novelty condition: the technology must display “innovative characteristics” or be of an “innovative nature”.
2. The substantial benefits condition: the innovative nature of the technology must bring substantial health benefits to the patient, also referred to as a “‘step-change’ in the management of the condition” [30].
3. The demonstrable and uncounted benefits condition: the substantial benefits brought by the innovative characteristics of the health technology must not already be captured in the incremental cost-effectiveness ratio (ICER) calculation of the technology under scrutiny and they must be “demonstrable and distinctive”.

If a health technology is judged to be innovative this might justify recommending a health technology for use in the NHS with an ICER greater than £20,000/Quality Adjusted Life Year (QALY) [29].

In April 2017 AIFA implemented a new system to define and measure drug innovation [15]. The new system judges the innovativeness of a new medicine on the basis of three indicators: the level of therapeutic need that the new drug is responding to, the added therapeutic value of the new medicine compared current practice, and the quality of the clinical evidence available to support the claims of benefit of the new intervention (assessed using the Grading of Recommendations Assessment, Development and Evaluation (GRADE) methodology [15]). The result can be one of three levels of innovative status: fully innovative, conditionally innovative or non-innovative. The process of reaching a conclusion about the level of innovativeness of a new drug has a deliberative component, whereby the components of the Scientific and Technical Committee (Commissione Tecnico-Scientifica, CTS) assign a level to each one of the 3 indicators of innovativeness, and then discuss the overall level of innovative status appropriate for each new drug. Depending on the level of innovativeness obtained, a new drug might benefit from access to the so-called innovative drug fund and/or immediate inclusion in regional formularies, avoiding that way any re-assessments at the regional/local level. These forms of assessment coupled with incentives for chosen technologies are meant to accelerate access to therapies deemed as innovative in the Italian healthcare system.

In France, HAS evaluates medicines and other health technologies. It considers innovation as the improvement in expected benefit (IEB) [31], taking account of the improvement in efficacy and/or safety brought by the new technology compared to others available with the same indication. Other dimensions that contribute to define innovation are taken into account in their assessment of actual clinical benefit (ACB). ACB includes the severity of the disease and the ‘public health benefit’. Public health benefit includes organizational dimensions, economic outcomes and the impact on the state of health of the population. The ACB is not comparative and it is used to determine if the new technology assessed should be reimbursed or not, while prices are negotiated on the basis of the IEB [24]. Secondary criteria for evaluating the degree of innovation include discerning between symptomatic, preventive and curative, and, for medical devices and medical equipment, HAS takes account of how disruptive the new technology is (that ‘affect existing technologies in the health field, and that may definitely replace them’) in contrast to others that might just be incrementally innovative (that only ‘show technological improvement in comparison with other devices’) [25]. However, there are no mechanisms in place specifically to reward innovations that suppose a disruptive change. There are only access-with-evidence-development schemes for devices that did not show sufficient ACB but were deemed to be of promising innovative value. Additionally, to reward innovative medicines appropriately while still collecting evidence HAS recently published their ‘Innovative medicines assessment action plan’, which expands the remit of conditional access schemes, reinforcing the use of real-world evidence to monitor medicines that have entered the market with high levels of uncertainty, fast-tracking access to promising therapies amongst other measures to better support innovation, along with other improvements in their processes [32].

In Japan, the Ministry of Health, Labour and Welfare generally reimburses all drugs and devices recommended by the Japanese regulatory agency. Pricing decisions for new health technologies are made by that same ministry but the NIPH, supported by various academic groups, coordinates the review process of the evidence submitted by manufacturers in their reimbursement applications [27]. Innovation is rewarded using a premium system, whereby new health technologies considered to be innovative are priced between 5 and 120% beyond the price of the comparator. The size of the premium is decided based on the number of the following criteria met by the new technology: (i) new mechanism of action; (ii) higher safety or efficacy; (iii) improvement of treatment for target disease, and; (iv) beneficial presentation [27].

In Spain the criteria that should be taken into account to decide whether a medicine is reimbursed by the National Healthcare System (NHS) are [16]: a) severity of the disease; b) the specific needs of certain groups of people; c) the therapeutic and social value of the medicine and incremental clinical benefit taking into account its cost-effectiveness; d) the rational use of public expenditure and the budget impact to the health service; e) the existence of therapeutic alternatives at lower price; and f) the degree of innovation of the medicine. In theory, decisions to include new medicines in the basic package covered by the National Health System, which sit with the CIPM, are made taking into account those criteria. However, the law does not define these terms or regulate how they are to be used, weighted or combined in decision-making. HTA reports also include data on safety and other factors as deemed relevant [22], but these attributes are not specifically mentioned in the P&R legislation. Despite the degree of innovation being amongst the criteria formally required for reimbursement of new medicines in Spain since 2006 [33], there is currently no definition of the concept in the public domain, nor is there a commonly accepted methodology to measure it.

For non-health technologies, the Spanish Network of Agencies for Health Technology Assessment and Services of the National Health System (RedETS) and GuíaSalud coordinate the HTA activities of the regional agencies and units in Spain and their guideline producing activities respectively, working towards the harmonization of methods applied in Spain for the assessment of health technologies and their inclusion in clinical guidelines. There are no official guidelines for how to price or reimburse non-pharmaceutical technologies. However, REdETS has published a ‘guideline for the elaboration and adaptation of rapid HTA reports’ [34], which outlines the dimensions taken into account also in full HTAs of non-drug health technologies in Spain. That is: safety, efficacy (within this efficacy dimension, there is a sub-section that captures what they refer to as patient satisfaction and acceptability), implementation considerations (economic – budget impact and efficiency of the technology –, organizational, and ethical, social and legal). This suggests that in Spain in practice, broadly speaking, similar criteria are used for medicines and non-drug health technologies, although importantly innovation is not mentioned amongst the criteria considered for non-drug health technologies.

It is worthwhile highlighting that, besides incentivizing companies to innovate by rewarding them pricing favorably and purchasing the innovations they bring to the market, states do also reward innovative companies with fiscal benefits. For instance, Spain has what they call Profarma, which is a program to stimulate the pharmaceutical sector in Spain incentivizing innovative companies with fiscal incentives. The aim is, mainly, to incentivize companies to invest in Spain, for instance setting up production and/or R&D centers there [35].

## Discussion

### Attributes of innovation that may be used as criteria for HTA decisions

The countries analysed here can be divided into 2 groups with respect to how they define innovation. France, Japan and Italy use features such as severity, unmet need and therapeutic added value as indicators of the degree of innovation of a health technology, while England, Spain consider the degree of innovation as a separate and additional criterion from others. However, official methodological guidelines in England or Spain do not offer much guidance as to how decision makers should measure innovation, leaving such matters to the discretion of the committee members.

Hence for countries such as Spain that aim to evaluate the degree of innovation as a separate criterion, it is worthwhile to offer some clarity about which attributes of the technology are being measured. This section applies the framework of Diaby and Goeree [14] to whittle down the items identified in the literature review to a set of attributes related to innovation that could be used as criteria for HTA in the countries of interest. Spain is taken as a “case study”, though the general approach is meant to be generalisable to other jurisdictions.

A comprehensive’ set of decision-making criteria would encompass all the dimensions listed in Table 2. The legislation in Spain does not mention step-change’, convenience’, strength of evidence base’ or impact on future R&D’ as criteria. This does not mean these items are ignored in HTA in Spain, only that they are not explicitly listed, and so we take these dimensions forward as candidates for inclusion in the category of ‘innovation’ for Spain. A comprehensive set of criteria would also be applicable to both medicines and other technologies. In some cases this can be achieved by tweaking the definition. For example, novelty refers to new drug structures or mechanisms of action, but it could very well refer to innovative mechanical architectures in the case of a device.

‘Value relevance’ refers in this context to whether a particular candidate item reflects the preferences of decision-makers with regard to the level of innovation in a product. Decisions makers in each jurisdiction would have to judge whether a given item is relevant and important to the decision problem at hand.

‘Non-redundancy’ refers to whether criteria are all necessary and do not repeat, double-count or overlap. NICE recognise this by requiring that benefits brought by the innovative characteristics of the health technology must not already be captured in other dimensions. For example, if the novel mode of administration leads to better adherence and hence greater effectiveness, this benefit should not be double-counted both in `added therapeutic value’ and in ‘patient convenience’. ‘Independence’ requires that the items are mutually exclusive, such that the level of performance in one item does not influence assessments about others.

Decision-makers must have a common understanding of what the criteria aim to measure to achieve precision and legitimacy. The items in Table 2 seem mostly self-explanatory, possibly with the exception of spill-over effects and real-option value. These items are rather abstract and might require explanation for decision-makers.

‘Measurement’ of each item does not have to be necessarily quantitative, but must be sufficiently rigourous and reproducible to avoid bias and achieve a reasonable degree of precision. Decision makers in HTA already have tools for measuring some of the items that might constitute a criterion of innovation. Where products promise a ‘step-change’, regulators (e.g. the Food & Drug Administration (FDA) in the United States, or European Medicines Agency (EMA) in Europe) may enable priority designation policies and accelerated access pathways, for devices [36], therapies generally [37, 38] and for specific cases such as gene therapies [39]. The strength of the evidence base is commonly assessed by applying a hierarchy of evidence [40] and where relevant might also capture uncertainties related to the learning curve [41]. There are a variety of instruments and outcome measures for patient convenience, though these are not comprehensive or easily transferable between patient groups or technology types. There is some theoretical work on how real option value might be measured, though it has yet to be validated in practice [42]. Spill-over would be challenging to measure as a HTA criteria.

### Towards a concept of innovation in Spain

The concept of innovation in healthcare has been widely described and discussed in the literature. However, rarely has it been done thinking about how different countries could go about defining a concept that fits with their HTA systems, to then be able to measure it and incorporate it in their methods guides and their assessments of different types of health technologies. It has been argued in the past that, although there might be distinct features of innovation worth rewarding distinctively, it would only be advisable to do so if innovation can be defined clearly and distinctively enough from other value dimensions already accounted for in the system, and if sustainable ways of rewarding innovativeness can be devised [43]. In this paper, the use of a case study allows us to point to how this concept might be tailored to a particular HTA system.

Our findings suggest that the following dimensions might be candidates for a criterion of innovation, at least in the context of HTA in Spain: ‘step-change’, ‘convenience’, ‘strength of evidence base’ and ‘impact on future R&D′. Of these, the concepts of step-change and strength of evidence base appear to be most straightforward to measure using existing instruments and procedures. However, in the context of innovative technologies, they are in some instances not entirely mutually exclusive. For ‘step-change’, regulators have designations such as ‘breakthrough’, and ‘fast-track’ that indicate serious conditions with a potential for significant improvement or unmet need, but at the same time high uncertainty. The evidence base may be undeveloped or weak, leading regulators to require further evidence collection as a condition of approval. HTA decision makers may also wish to stipulate further evidentiary or conditional reimbursement conditions for adoption into national health systems. The relevance of items such as ‘convenience’ or ‘novel mode of administration’ depends on context, though it is important to avoid double counting benefits and to apply such criteria consistently across different indications and interventions. Undoubtedly the most abstract and difficult to measure are items related to the interaction between current adoption decisions and the direction of future R&D. Novelty per-se might be seen as a necessary but not sufficient condition for recognising a technology as innovative, apart from specific circumstances such as an option for patients who are contra-indicated for existing interventions. Real –option value also would only be applicable in very specific circumstances, where patients need to buy time until they can take advantage of another new therapy just on the horizon. Scientific spillover effects are quite abstract and diffuse. R&D investment is a global enterprise influenced by a multitude of factors, and HTA decision-making procedures in individual countries and individual indications may have only a marginal impact, if any. However, there may be specific contexts where scientific advance is propelled forward by synergistic achievements in related areas, such as gene or cell therapies, and this might be usefully recognised at national level.

A change in HTA criteria requires transparency, robustness and an integrative process that gives the opportunity to different stakeholders to present their perspectives [44]. MCDA could and has been used to measure the degree of innovation [18, 45–52], to weight the different items to produce an overall innovation score and/or weight the importance of innovation relative to other value dimensions. However, it can be a complex method, data hungry and challenging to use routinely adhering to good practice guidelines [53], particularly by smaller HTA bodies, though the challenges are not insurmountable. A more pragmatic approach could be the use of a checklist, which is something that has already been done for other purposes in HTA [54].

Research into the extent to which innovation is actually captured and used in practice in decision making in HTA suggests that it is indeed taken into account in decision making, and in fact it is referred to by NICE with a high frequency relative to other criteria in their appraisal documents [55]. However, it does not rank between the most relevant criteria for most decision makers from across the world [56]. An interesting step further would be to explore the societal (i.e. public’s) preferences for innovation [57] in any country considering its inclusion in their HTA systems.

## Conclusions

If innovation is to be used as operational criteria for adoption and P&R of health technologies, the concept must be clearly defined, and it ought to be independent from other value dimensions already captured in HTA systems. We acknowledge that, in the present paper, we have only superficially touched upon these ways of enabling innovation in health technology assessment, and further research would be to work with decision makers to produce a practical framework.

## Supplementary Information

**Additional file 1.** Annex I – Search Strategy

**Additional file 2.** Annex II – Brief summaries of included papers

## Data Availability

All data generated or analysed during this study are included in this published article [and its supplementary information files].

## Abbreviations

ACB: Actual Clinical Benefit
AIFA: Agenzia Italiana del Farmaco
CTS: Commissione Tecnico-Scientifica
EMA: European Medicines Agency
FDA: Food & Drug Administration
GRADE: Grading of Recommendations Assessment, Development and Evaluation
HAS: Haute Autorité de Santé
HTA: Health Technology Assessment
IEB: Improvement in Expected Benefit
ICER: Incremental Cost-Effectiveness Ratio
CIPM: Interministerial Medicinal Products Pricing Committee
MCDA: Multiple Criteria Decision Analysis
NHS: National Healthcare System
NICE: National Institute for Health and Care Excellence
NIPH: National Institute of Public Health
P&R: Pricing & Reimbursement
QALY: Quality Adjusted Life Year
R&D: Research and Development
AEMPS: Spanish Agency of Medicines and Medical Devices
RedETS: Spanish Network of Agencies for Health Technology Assessment and Services of the National Health System
WHO: World Health Organization
