# Supplementary material for: How innovation can be defined, evaluated and rewarded in health technology assessment
Source: Health Econ Rev. 2022 Jan 3;12:1. doi: 10.1186/s13561-021-00342-y (PMC8725438; doi:10.1186/s13561-021-00342-y)
Supplement: Supplementary file 1 — Additional file 1. Annex I – Search Strategy [file 13561_2021_342_MOESM1_ESM.docx]

Annex I – Search Strategy

To search for relevant literature we used Medline (Ovid) and Google Scholar using free search terms including innovation or Invention or improvement, assessing or defining or measuring or value or degree or technological innovation, Industry or Discovery or Investigation or Design or Evaluation or cost or approval drug, cost or ‘cost benefit analysis' or 'health care costs', assessment technology and MESH terms including Innovation or "Diffusion of Innovation" or Inventions, Cost-Benefit Analysis, Biomedical Technology or Technology, Pharmaceutical, Technology Assessment, Biomedical. These search terms were combined using Boolean terms and proximity operators. These searches allowed us to identify the 4 seminal papers that form the basis of our snowballing search. If, whilst searching for those 4 seminal papers, we found papers we found relevant for our review but that did not quite meet our requirements to become one of our seminal papers, we included them in the review as a standard reference. We also run manual secondary searches within the lists of references of the included papers to identify additional relevant studies, performing the snowballing search technique described in the methods section of this paper. We limited the search to publications in Spanish or English. We imposed no limitations to the date of publication of the manuscripts. We selected and included additional publications for this review on the basis of our expertise in the field of health technology assessment, and in particular in the topic of innovation related to health policy. We also added additional relevant records to our search through recommendations of colleagues and through contacts within our networks with expertise in the topic under study in this paper, as suggested by Greenhalgh & Peacock (2015) in their paper (see reference 13 in the main body of our paper).
