# Supplementary material for: How innovation can be defined, evaluated and rewarded in health technology assessment
Source: Health Econ Rev. 2022 Jan 3;12:1. doi: 10.1186/s13561-021-00342-y (PMC8725438; doi:10.1186/s13561-021-00342-y)
Supplement: Supplementary file 2 — Additional file 2. Annex II – Brief summaries of included papers [file 13561_2021_342_MOESM2_ESM.docx]

Annex II – Brief summaries of included papers

| Nr. | Reference | Brief Summary |
| --- | --- | --- |
| 1 | Mestre-Ferrandiz J, Sussex J, Towse A. The R&D cost of a new medicine. London: Office of Health Economics; 2012. | This publication reviews research published over the last three decades on the cost of R&D for a successful new medicine, and explores the major factors that are leading to higher R&D costs. |
| 2 | DiMasi JA, Grabowski HG, Hansen RW. Innovation in the pharmaceutical industry: New estimates of R&D costs. J Health Econ. 2016;47:20-33. doi:10.1016/j.jhealeco.2016.01.012. | This study presents the research and development costs of 106 randomly selected new drugs obtained from a survey of 10 pharmaceutical firms. They used these data to estimate the average pre-tax cost of new drug and biologics development. |
| 3 | World Health Organization. Health Technology Assessment. [Internet] Geneva WHO; [2021] [cited 2021 3 jan]; Available from: <https://www.who.int/health-technology-assessment/about/en/>. | WHO website page defining Health Technology Assessment (HTA). |
| 4 | Claxton K. OFT, VBP: QED? Health Econ. 2007;16:545-58. doi:10.1002/hec.1249. | Critique examining the theoretical underpinnings of the report by the British Office of Fair Trading on the UK pharmaceutical price regulation scheme (PPRS) published in 2007. |
| 5 | de Solà-Morales O, Cunningham D, Flume M, Overton PM, Shalet N, Capri S. Defining innovation with respect to new medicines: a systematic review from a payer perspective. Int J Technol Assess Health Care. 2018;34:224-40. doi:10.1017/s0266462318000259. | Systematic literature review investigating how innovation is defined with respect to new medicines and assessing the extent to which published definitions incorporate the impact of new medicines on healthcare costs. |
| 6 | Claxton K, Martin S, Soares M, Rice N, Spackman E, Hinde S, et al. Methods for the estimation of the National Institute for Health and Care Excellence cost-effectiveness threshold. Health Technol Assess. 2015;19:1-503, v-vi. doi:10.3310/hta19140. | This paper presents the conceptual and methodological framework that builds the cost-effectiveness threshold used by NICE, and presents a best estimate of the threshold for policy purposes. |
| 7 | Ciani O, Armeni P, Boscolo PR, Cavazza M, Jommi C, Tarricone R. De innovatione: The concept of innovation for medical technologies and its implications for healthcare policy-making. Health Policy Technol. 2016;5:47-64. doi:10.1016/j.hlpt.2015.10.005. | This systematic literature review of the academic literature aims to summarise acceptable definitions of innovation in relation to medical devices. |
| 8 | Ilinca S, Hamer S, Botje D, Espin J, Veloso Mendes R, Müller J, et al. All You need to know about innovation in healthcare: The best 10 reads. Int J Healthc Manag. 2012;5:193-202. doi:10.1179/2047971912y.0000000018. | Study presenting the results of a Delphi panel conducted to identify and select the 10 most relevant and informative scientific writings, which can add significantly to the knowledge of managers by offering an introduction in the academic discussion on the topic of innovation in healthcare. |
| 9 | Juárez Castelló CA, Antoñanzas Villar F, Pinillos García MO. Innovación en medicamentos: efectos para el" cliente" público y cambios legislativos recientes. In: Ayala Calvo JC, Universidad de la Rioja, Grupo de Investigación Fedra, editors. Conocimiento, innovación y emprendedores : camino al futuro. Logroño: Universidad de la Rioja; [2007]. p. 1466-81. | This paper presents an analysis of the R&D process of medicines in the context of the degree and types of innovation they bring by. Additionally, the authors review the main economic aspects of the 2006 Act of Guarantees and Rational Use of the Medicine and the Sanitary Products in Spain. |
| 10 | Motola D, De Ponti F, Rossi P, Martini N, Montanaro N. Therapeutic innovation in the European Union: analysis of the drugs approved by the EMEA between 1995 and 2003. Br J Clin Pharmacol. 2005;59:475-8. doi:10.1111/j.1365-2125.2004.02320.x. | The authors review the list of drugs approved by the EMEA between January 1995 through the first 6 months of 2003, and assign to them scores for therapeutic innovation assigned through a consensus process classifying them into one of three degrees of innovation: ‘A’ (important), ‘B’ (moderate) and ‘C’ (modest). |
| 11 | Moreno SG, Ray JA. The value of innovation under value-based pricing. J Mark Access Health Policy. 2016;4. doi:10.3402/jmahp.v4.30754. | In this paper, the authors outline the limitations of the conventional cost-effectiveness analysis approach, while proposing an alternative method of evaluation that, they argue, captures the value of innovation more accurately. |
| 12 | Badampudi D, Wohlin C, Petersen K. Experiences from Using Snowballing and Database Searches in Systematic Literature Studies. Proceedings 19th International Conference on Evaluation and Assessment in Software Engineering (EASE 2015), Nanjing, China; 2015: ACM Press; 2015. | This study evaluates the efficiency and reliability of snowballing search techniques when used as a search strategy in literature studies. They also compare the performance of snowballing searches with database searches. |
| 13 | Greenhalgh T, Peacock R. Effectiveness and efficiency of search methods in systematic reviews of complex evidence: audit of primary sources. Bmj. 2005;331(7524):1064-5. Greenhalgh T, Peacock R. Effectiveness and efficiency of search methods in systematic reviews of complex evidence: audit of primary sources. Bmj. 2005;331(7524):1064-5. doi:10.1136/bmj.38636.593461.68. | This paper is a review of reviews, aimed at describing where papers come from in systematic reviews of complex evidence. The authors assess whether formal protocol-driven search strategies are sufficient to respond complex policy questions, or if other searching techniques may perform better. |
| 14 | Diaby V, Goeree R. How to use multi-criteria decision analysis methods for reimbursement decision-making in healthcare: a step-by-step guide. Expert Rev Pharmacoecon Outcomes Res. 2014;14:81-99. doi:10.1586/14737167.2014.859525. | This article presents the main MCDA decision support methods (elementary methods, value-based measurement models, goal programming models and outranking models) using a case study approach. The authors provide a step-by-step guide on how to use MCDA methods for reimbursement decision-making in healthcare. |
| 15 | Fortinguerra F, Tafuri G, Trotta F, Addis A. Using GRADE methodology to assess innovation of new medicinal products in Italy. Br J Clin Pharmacol. 2020;86:93-105. doi:10.1111/bcp.14138. | The aim of this study is to describe the new model that the Italian Medicine Agency (AIFA) presented in April 2017 to grant any new medicinal product with an innovative designation. |
| 16 | Real Decreto Legislativo 1/2015 de 24 de julio. Ley de garantías y uso racional de los medicamentos y productos sanitarios., Boleín Oficial del Estado, nº 177, (25-07-2015). | Legislative Royal Decree, published in July 2015, whereby a series of previous legislative measures are consolidated into one law of guarantees and rational use of medicines and health products. |
| 17 | Garrison LP, Jr., Kamal-Bahl S, Towse A. Toward a Broader Concept of Value: Identifying and Defining Elements for an Expanded Cost-Effectiveness Analysis. Value Health. 2017;20:213-16. doi:10.1016/j.jval.2016.12.005. | This paper identifies and defines potentially useful expansions to traditional cost-effectiveness analysis as often used in health technology assessment. The authors propose a an expanded framework, incorporating a wider range of elements of value to health technology assessment. |
| 18 | Angelis A, Kanavos P. Multiple Criteria Decision Analysis (MCDA) for evaluating new medicines in Health Technology Assessment and beyond: The Advance Value Framework. Soc Sci Med. 2017;188:137-56. doi:10.1016/j.socscimed.2017.06.024. | This study proposes a Multiple Criteria Decision Analysis (MCDA) methodological process for the assessment of medicines, eliciting 5 key domains of value and structuring them into a generic value tree. The combination of these MCDA modelling techniques for the elicitation and construction of value preferences across a generic value tree provides a new value framework (Advance Value Framework) enabling the comprehensive measurement of value in a structured and transparent way. |
| 19 | Moher D, Liberati A, Tetzlaff J, Altman DG. Preferred reporting items for systematic reviews and meta-analyses: the PRISMA statement. BMJ. 2009;339:b2535. doi:10.1136/bmj.b2535. | In 1996, an international group developed the QUOROM Statement (Quality Of Reporting Of Meta-analyses), which focused on the reporting of meta-analyses of randomized controlled trials. This paper summarises a revision of these guidelines, renamed PRISMA (Preferred Reporting Items for Systematic reviews and Meta-Analyses), updated to address several conceptual and practical advances in the science of systematic reviews. |
| 20 | Mestre-Ferrandiz J, Mordoh A, Sussex J. The many faces of innovation. A report for the ABPI by the Office of Health Economics. London: Association of the British Pharmaceutical Industry; 2012. | The objective of this report is to aid understanding of the nature of innovation in the pharmaceutical industry. To do so, they do a review of the literature on the economics of innovation and present a series of case studies of several therapy areas to demonstrate pharmaceutical innovation in practice showing cases where there were major advances in treatments bringing by innovation to the market. |
| 21 | Kennedy I. Appraising the Value of Innovation and Other Benefits: A Short Study for NICE. [Internet] London: NICE; 2009 [cited 2021 23 feb]; Available from: <https://www.nice.org.uk/Media/Default/About/what-we-do/Research-and-development/Kennedy-study-final-report.pdf>. | This report is a result of NICE commissioning a study to respond to the following questions: (i) Are there any benefits (or values) which NICE should take into account in its technology appraisals which it currently does not capture?; (ii) is innovation as a benefit properly taken account of?; (iii) to the extent that innovation and other benefits should be taken into account, how should NICE do so? |
| 22 | Charlton V, Rid A. Innovation as a value in healthcare priority-setting: the UK experience. Soc Justice Res. 2019;32:208-38. doi:10.1007/s11211-019-00333-9. | The authors use UK’s NICE as an example to examine how efforts to promote healthcare innovation in the priority-setting process can play part in the inevitable trade-offs between maximising health and promoting health equity. They nalyse under what conditions NICE recommends funding technologies of an “innovative nature”, even when these technologies do not satisfy NICE’s cost-effectiveness criteria. |
| 23 | National Institute for Health and Care Excellence. Single technology appraisal: User guide for company evidence submission template. [Internet] London: NICE; 2015 [cited 2021 18 feb]; Available from: <https://www.nice.org.uk/process/pmg24/resources/single-technology-appraisal-user-guide-for-company-evidence-submission-template-pdf-72286715419333>. | This is the user guide for submission of evidence to the National Institute for Health and Care Excellence (NICE) as part of the single technology appraisal (STA) process. It explains what information NICE requires and the format in which it should be presented. |
| 24 | Haute Autorité de Santé. Annual report 2005. [Internet] Saint-Denis La Plaine: HAS; 2005 [cited 2021 1 jan]; Available from: <https://www.has-sante.fr/upload/docs/application/pdf/ra_gb_has_2005.pdf>. | This annual report presents the activities carried out by HAS in 2005 and looks ahead into the future plans of the agency. |
| 25 | Haute Autorité de Santé. Pricing & Reimbursement of drugs and HTA policies in France. Saint-Denis La Plaine: HAS; 2014 [cited 2021 2 jun]; Available from: <https://www.has-sante.fr/upload/docs/application/pdf/2014-03/pricing_reimbursement_of_drugs_and_hta_policies_in_france.pdf>. | This is a presentation outlining the reimbursement and pricing system for drugs in place in France, describing the aspects of value of a drug considered by HAS and how those are incorporated in a system to make reimbursement and pricing decisions. It also describes more procedural aspects of the system. |
| 26 | Dubromel A, Geffroy L, Aulagner G, Dussart C. Assessment and diffusion of medical innovations in France: an overview. J Mark Access Health Policy. 2018;6:1458575. doi:10.1080/20016689.2018.1458575. | This article provides an overview of the assessment and diffusion of medical innovation in France. The authors also discuss key opportunities and challenges of medical innovation assessment and diffusion in France. |
| 27 | Haute Autorité de Santé. Innovative medicines assessment action plan [Internet] Saint-Denis La Plaine: HAS; 2020 [cited 2021 22 feb]; Available from: <https://www.has-sante.fr/upload/docs/application/pdf/2020-03/innovative_medicine_action_plan_27.01.20.pdf>. | This report presents HAS’ 2020 action plan for innovative therapies, describing their plans to respond to an environment where increasingly drugs reach HTA agencies with evidence that presents numerous unresolved uncertainties, most notably in cases of short clinical development often seen for small patient sample sizes. |
| 28 | Ministerio de Sanidad Servicios Sociales e Igualdad. Propuesta de colaboración para la elaboración de los informes de posicionamiento terapéutico de los medicamentos. [Internet] Madrid: AEMPS; 2013 [cited 2021 19 feb]; Available from: <https://www.aemps.gob.es/medicamentosUsoHumano/informesPublicos/docs/propuesta-colaboracion-informes-posicionamiento-terapeutico.pdf>. | This document sets the foundations for a collaborative process to produce the therapeutic positioning reports for medicines in Spain. It describes the procedure and agents involved in the production of these reports, and it also describes the phases in which the reports will be generated. |
| 29 | Ley 29/2006, de 26 de julio. Ley de garantías y uso racional de los medicamentos y productos sanitarios, Boleín Oficial del Estado, nº 178, (27-07-2006) (2006). | Law for the rational use of medicines and health products, published in 2006, which regulates the reimbursement and financing mechanisms for these products in Spain. |
| 30 | Puñal-Riobóo J, Baños Álvarez E, Varela Lema L, Castillo Muñoz MA, Atienza Merino G, Ubago Pérez R, et al. Guía para la elaboración y adaptación de informes rápidos de evaluación de tecnologías sanitarias. Madrid. Santiago de Compostela: Red Española de Agencias de Evaluación de Tecnologías Sanitarias y Prestaciones del SNS. Agencia Gallega para la Gestión del Conocimiento en Salud. Unidad de Asesoramiento Científico-técnico, Avalia-t; 2016. | Methodological document describing the methodology used by the Spanish Network of Health Technology Assessment Agencies in their assessments. The Network seeks a unified and coordinated approach to the production of HTAs of medical devices in Spain. |
| 31 | National Institute for Health and Care Excellence. Guide to the methods of technology appraisal 2013. [Internet] London: NICE; 2013 [cited 2021 19 feb]; Available from: <https://www.nice.org.uk/process/pmg9/resources/guide-to-the-methods-of-technology-appraisal-2013-pdf-2007975843781>. | This document provides an overview of the principles and methods of health technology assessment and appraisal within the NICE technology appraisal process. |
| 32 | Angelis A, Lange A, Kanavos P. Using health technology assessment to assess the value of new medicines: results of a systematic review and expert consultation across eight European countries. Eur J Health Econ. 2018;19:123-52. doi:10.1007/s10198-017-0871-0. | In this paper, the authors study the practices, processes and policies of value-assessment for new medicines across eight European countries and the role of HTA beyond economic evaluation and clinical benefit assessment. The countries under study in this article are France, Germany, England, Sweden, Italy, Netherlands, Poland and Spain. |
| 33 | Epstein D, Espín J. Evaluation of new medicines in Spain and comparison with other European countries. Gac Sanit. 2020;34:133-40. doi:10.1016/j.gaceta.2019.02.009. | The authors of this study compare the use of HTA as a tool to support pricing and reimbursement (P&R) of new medicines in Spain with its use in England, Sweden, France and Germany. |
| 34 | Kamae I, Thwaites R, Hamada A, Fernandez JL. Health technology assessment in Japan: a work in progress. Journal of medical economics. 2020;23(4):317-22. doi:10.1080/13696998.2020.1716775. | This paper provides an update on recent HTA developments in Japan and key challenges still to be addressed, reporting the results of a review of publications and commentaries since April 2019, together with views from a group of experts on key issues to be addressed. |
| 35 | Shiroiwa T, Fukuda T, Ikeda S, Takura T. New decision-making processes for the pricing of health technologies in Japan: The FY 2016/2017 pilot phase for the introduction of economic evaluations. Health Policy. 2017;121(8):836-41. doi:10.1016/j.healthpol.2017.06.001. | In this paper, the authors provide an overview of relevant discussions and the process of trial implementation of the reformed Japanese system after a brief explanation of the Japanese system of pricing drugs and medical devices. |
| 36 | Ministerio de Industria Comercio y Turismo. PROFARMA (2017-2020): Fomento de la competitividad en la Industria Farmacéutica. [Internet] Madrid: Ministerio de Industria, Comercio y Turismo; 2017 [cited 2021 16 feb]; Available from: <https://www.mincotur.gob.es/PortalAyudas/profarma/Descripcion/Paginas/objetivos.aspx>. | This is the webpage within the Spanish Ministry of Industry describing PROFARMA, a government lead programme aiming to incentivise the pharmaceutical sector to invest in Spain. To do so, they empower pharmaceutical companies to invest in new production plants in the country, invest in new manufacturing technologies and facilities in Spain. Their incentives are mainly fiscal. |
| 37 | Food and Drug Administration. Breakthrough Devices Program. [Internet] Silver Spring: FDA; 2021 [cited 2021 22 jan]; Available from: <https://www.fda.gov/medical-devices/how-study-and-market-your-device/breakthrough-devices-program>. | FDA webpage presenting their breakthrough devices program. It is a is a voluntary program for certain medical devices and device-led combination products that provide for more effective treatment or diagnosis of life-threatening or irreversibly debilitating diseases or conditions. It replaces their earlier Expedited Access Pathway and Priority Review for medical devices. |
| 38 | Food and Drug Administration. Breakthrough Therapy. [Internet] Silver Spring: FDA; 2018 [cited 2021 24 jan]; Available from: <https://www.fda.gov/patients/fast-track-breakthrough-therapy-accelerated-approval-priority-review/breakthrough-therapy>. | FDA webpage presenting their breakthrough therapy program, which is a process designed to expedite the development and review of drugs that are intended to treat a serious condition and preliminary clinical evidence indicates that the drug may demonstrate substantial improvement over available therapy on a clinically significant endpoint(s). |
| 39 | Baird LG, Banken R, Eichler HG, Kristensen FB, Lee DK, Lim JC, et al. Accelerated access to innovative medicines for patients in need. Clin Pharmacol Ther. 2014;96:559-71. doi:10.1038/clpt.2014.145. | This paper describes the specific approaches that have been taken in four economically developed regions to the design and implementation of early-access pathways or initiatives, reviews their success rates, and suggests possible new directions. |
| 40 | Yitong Wang TQ, Shuyao Liang, Claude Dussart. Regulatory Pathways in Europe, the United States, and Japan and Health Technology Assessments for Gene Therapies. Value & Outcomes Spotlight. 2020;6:37-41. | This article given reviews the marketing-authorization pathways for cell and gene therapies in the European Union (EU), the United States, and Japan. Furthermore, the authors compared the regulatory and reimbursement status of gene therapies in the United States and 5 European countries: France, the United Kingdom (England and Scotland), Germany, Italy, and Spain. |
| 41 | Guyatt GH, Oxman AD, Vist GE, Kunz R, Falck-Ytter Y, Alonso-Coello P, et al. GRADE: an emerging consensus on rating quality of evidence and strength of recommendations. BMJ. 2008;336:924-6. doi:10.1136/bmj.39489.470347.AD. | This paper explores the advantages of the GRADE system, which is increasingly being adopted by organisations all over the world, to help reduce inconsistencies in how guideline developers worldwide rate the quality of evidence and the strength of recommendations. |
| 42 | Ramsay CR, Grant AM, Wallace SA, Garthwaite PH, Monk AF, Russell IT. Assessment of the learning curve in health technologies. A systematic review. Int J Technol Assess Health Care. 2000;16:1095-108. doi:10.1017/s0266462300103149. | The authors of this work reviewed and appraised the methods by which the issue of the learning curve has been addressed during health technology assessment in the past. |
| 43 | Thornton Snider J, Romley JA, Vogt WB, Philipson TJ. The Option Value of Innovation. Forum Health Econ Policy. 2012;15. doi:10.1515/1558-9544.1306. | This paper defines the term “option value” of innovation in health technologies, explain how to calculate it in a variety of standard cost effectiveness analysis contexts and provide a proof-of-concept using the example of the drug tamoxifen. |
| 44 | Ferner RE, Hughes DA, Aronson JK. NICE and new: appraising innovation. BMJ. 2010;340:b5493. doi:10.1136/bmj.b5493. | In this article the authors consider how innovativeness might be defined in health care, and how NICE and other organisations analysing health technologies might allow it to influence appraisal decisions. |
| 45 | National Institute for Health and Care Excellence. NICE’s methods of technology evaluation - presenting a case for change. [Internet] London: NICE; 2020 [cited 2021 27 jan]; Available from: <https://www.nice.org.uk/news/article/nice-s-methods-of-technology-evaluation-presenting-a-case-for-change>. | Webpage within NICE’s website announcing the launch, on the 6 November 2020, of a public consultation on proposals for changes to the methods it uses to develop its guidance on medicines, medical devices and diagnostics. |
| 46 | Angelis A. Evaluating the Benefits of New Drugs in Health Technology Assessment Using Multiple Criteria Decision Analysis: A Case Study on Metastatic Prostate Cancer With the Dental and Pharmaceuticals Benefits Agency (TLV) in Sweden. MDM Policy & Practice. 2018;3:2381468318796218. doi:10.1177/2381468318796218. | The aim of this paper is to test in practice the Advance Value Framework, an MCDA methodological framework for HTA, in a proof-of-concept case study with decision makers from the Dental and Pharmaceuticals Benefits Agency (TLV) in Sweden. |
| 47 | Angelis A, Linch M, Montibeller G, Molina-Lopez T, Zawada A, Orzel K, et al. Multiple Criteria Decision Analysis for HTA across four EU Member States: Piloting the Advance Value Framework. Soc Sci Med. 2020;246:112595. doi:10.1016/j.socscimed.2019.112595. | This article presents the application of the Advance Value Framework (AVF), an MCDA methodology for HTA based on multi-attribute value theory, through a series of case studies with decision-makers in four countries (Sweden (TLV), Andalusia/Spain (AETSA), Poland (AOTMiT) and Belgium (INAMI-RIZIV)), to explore its feasibility and compare decision-makers' value preferences and results. |
| 48 | Angelis A, Thursz M, Ratziu V, O'Brien A, Serfaty L, Canbay A, et al. Early Health Technology Assessment during Nonalcoholic Steatohepatitis Drug Development: A Two-Round, Cross-Country, Multicriteria Decision Analysis. Med Decis Making. 2020;40:830-45. doi:10.1177/0272989x20940672. | The aim of this paper was to investigate the use of multicriteria decision analysis (MCDA) to support decision making during drug development while considering payer and health technology assessment (HTA) value concerns, by applying the Advance Value Framework in nonalcoholic steatohepatitis (NASH) and testing for the consistency of the results. |
| 49 | Baran-Kooiker A, Czech M, Kooiker C. Multi-Criteria Decision Analysis (MCDA) Models in Health Technology Assessment of Orphan Drugs-a Systematic Literature Review. Next Steps in Methodology Development? Front Public Health. 2018;6:287. doi:10.3389/fpubh.2018.00287. | This work provides an overview of the current state of the art and latest developments in the area of MCDA in HTA for orphan drugs, to review existing models, their characteristics, as well as to identify opportunities for further refinement. |
| 50 | Angelis A, Montibeller G, Hochhauser D, Kanavos P. Multiple criteria decision analysis in the context of health technology assessment: a simulation exercise on metastatic colorectal cancer with multiple stakeholders in the English setting. BMC Med Inform Decis Mak. 2017;17:149. doi:10.1186/s12911-017-0524-3. | This project tests in practice the Advance Value Framework (AVF), MCDA methodological framework, through a proof-of-concept case study in metastatic colorectal cancer engaging multiple stakeholders within the English setting. |
| 51 | Hsu JC, Lin JY, Lin PC, Lee YC. Comprehensive value assessment of drugs using a multi-criteria decision analysis: An example of targeted therapies for metastatic colorectal cancer treatment. PLoS One. 2019;14:e0225938. doi:10.1371/journal.pone.0225938. | This study paper presents a decision-making model with multiple criteria for appraisal and reimbursement to compare the attitudes of different stakeholders toward various dimensions and criteria and to evaluate the five targeted therapies (bevacizumab, cetuximab, panitumumab, aflibercept, and regorafenib) for metastatic colorectal cancer. |
| 52 | Jakab I, Németh B, Elezbawy B, Karadayı MA, Tozan H, Aydın S, et al. Potential Criteria for Frameworks to Support the Evaluation of Innovative Medicines in Upper Middle-Income Countries-A Systematic Literature Review on Value Frameworks and Multi-Criteria Decision Analyses. Front Pharmacol. 2020;11:1203-. doi:10.3389/fphar.2020.01203. | Systematic review aiming to facilitate the development of future MCDA frameworks, by proposing a set of criteria focusing on the purchasing decisions of single-source innovative pharmaceuticals in upper middle-income countries. |
| 53 | Thokala P, Duenas A. Multiple criteria decision analysis for health technology assessment. Value Health. 2012;15:1172-81. doi:10.1016/j.jval.2012.06.015. | This article analyses the possible application of MCDA approaches in health technology assessment and describes their relative advantages and disadvantages. |
| 54 | Phillips LD. Best Practice for MCDA in Healthcare. In: Marsh K., Goetghebeur M., Thokala P., Baltussen R, editors. Multi-Criteria Decision Analysis to Support Healthcare Decisions. Cham, Switzerland: Springer; 2017. p. 311-29. | Book chapter that presents the theoretical underpinnings of decision theory extending it to accommodate multiple criteria for evaluating the values of alternative courses of action, to then present an eight-step framework for constructing an MCDA model used to formulate best practice principles. |
| 55 | Hailey D. Toward transparency in health technology assessment: a checklist for HTA reports. Int J Technol Assess Health Care. 2003;19:1-7. doi:10.1017/s0266462303000011. | This paper presents an initiative of the International Network of Agencies for Health Technology Assessment (INAHTA) that developed a checklist for assessment reports as a means of improving transparency and consistency in HTA. |
| 56 | de Folter J, Trusheim M, Jonsson P, Garner S. Decision-components of NICE's technology appraisals assessment framework. Int J Technol Assess Health Care. 2018;34:163-71. doi:10.1017/s0266462318000090. | The authors present a novel application of text analysis that characterizes NICE's Technology Appraisals in the context of the newer assessment frameworks and they present the results in a visual way. They identify a hierarchical set of decision factors considered in the assessments, and determine the frequency of recurrence of decision factors. |
| 57 | Tanios N, Wagner M, Tony M, Baltussen R, van Til J, Rindress D, et al. Which criteria are considered in healthcare decisions? Insights from an international survey of policy and clinical decision makers. Int J Technol Assess Health Care. 2013;29:456-65. doi:10.1017/s0266462313000573. | This study gathers qualitative and quantitative data on criteria considered by healthcare decision makers from 23 countries in five continents. |
| 58 | Linley WG, Hughes DA. Societal views on NICE, cancer drugs fund and value-based pricing criteria for prioritising medicines: a cross-sectional survey of 4118 adults in Great Britain. Health Econ. 2013;22:948-64. doi:10.1002/hec.2872. | This paper explores societal preferences for criteria such as those used by the National Institute for Health and Clinical Excellence (NICE) for accepting higher incremental cost‐effectiveness ratios for some medicines over others, or to implement policies such as the Cancer Drugs fund and the attempt made to introduce the so called value based pricing scheme in England. To do so, they conducted a choice‐based experiment in 4118 UK adults via web‐based surveys. |
